# Annual of the Universal Medical Sciences

**Published:** 1892-10

**Authors:** 


### Book Reviews.

ANNUAL OF THE UNIVERSAL MEDICAL SCIENCES.—Edited by Chas. E. Sajous, M. D., and seventy associate editors, assisted by over two hundred corresponding editors, collaborators and correspondents. Five volumes. Published by the F. A. Davis Co., Philadelphia, 1892.

BUT few changes have been made in the general appearance and make-up of the fifth series from those of the preceding years. The general character of the articles, together with the corps of collaborators presented, indicate with what earnest endeavors the editors are striving to maintain that high degree of excellence which has characterized it since its first appearance. The Annual is yearly enlarging its scope of usefulness, and through the tireless devotion of its excellent editorial staff, among whom, we are pleased to note our colleague and fellow townsman, Prof. J. McFadden Gaston, who contributes a paper upon Thoracic Surgery, the publishers hope soon to witness its evolution into the state of perfection which has ever been its aim to reach.

